# *Drosophila* Models of Sporadic Parkinson’s Disease

**DOI:** 10.3390/ijms19113343

**Published:** 2018-10-26

**Authors:** Emi Nagoshi

**Affiliations:** Department of Genetics and Evolution, Sciences III, University of Geneva, 30 Quai Ernest-Ansermet, CH-1211 Geneva-4, Switzerland; Emi.Nagoshi@unige.ch

**Keywords:** sporadic Parkinson’s disease, *Drosophila*, animal model

## Abstract

Parkinson’s disease (PD) is the most common cause of movement disorders and is characterized by the progressive loss of dopaminergic neurons in the substantia nigra. It is increasingly recognized as a complex group of disorders presenting widely heterogeneous symptoms and pathology. With the exception of the rare monogenic forms, the majority of PD cases result from an interaction between multiple genetic and environmental risk factors. The search for these risk factors and the development of preclinical animal models are in progress, aiming to provide mechanistic insights into the pathogenesis of PD. This review summarizes the studies that capitalize on modeling sporadic (i.e., nonfamilial) PD using *Drosophila* *melanogaster* and discusses their methodologies, new findings, and future perspectives.

## 1. Introduction

Parkinson’s disease (PD) is the second most frequent neurodegenerative disorder and affects 7–10 million individuals worldwide. It presents with motor symptoms, such as bradykinesia, rigidity, postural instability and resting tremor; however, it also manifests a broad spectrum of nonmotor symptoms, including sleep disturbances, mood disorders, and cognitive impairments [1,2]. A prominent pathology of PD is the loss of dopamine (DA) neurons in the substantia nigra pars compacta (SNpc), which projects to the striatum. The resulting striatum DA deficiency leads to the characteristic motor dysfunction. Another histopathological hallmark of PD is the presence of abnormal aggregates (inclusions) that contain the α-synuclein (α-syn) protein, termed Lewy bodies and Lewy neurites [3], although many PD cases without Lewy body pathology have been reported [4,5]. No treatment strategy to cure or slow down PD progression is currently available. With a prevalence of approximately 1% at 60 years of age [6], a better understanding of PD pathogenesis for developing an improved treatment strategy is an increasing need in aging societies.

The role of genetics in the etiology of PD had long been debated as only approximately 15% of PD patients have a family history and no more than 10% of cases have Mendelian inheritance [1,7]. However, the identification of monogenic mutations that cause the Mendelian forms of PD has unequivocally placed genetic factors in the center of PD etiology. A positive family history can be explained by the exposure to common environmental factors, monogenic or polygenic inheritance, and a combination thereof [8]. Linkage studies of families with non-Mendelian inheritance of PD have provided evidence of gene-gene interaction as a PD risk factor in these families [9,10,11], which supports a genetic contribution to the non-monogenic familial PD.

PD cases that occur in individuals with no positive family history are defined as “sporadic PD”. However, this categorization does not exclude genetic factors as causative agents. It most likely has a multifactorial origin, which results from complex interactions of genetic and environmental factors. Sporadic PD can be monogenic because a dominant mutation may have occurred *de novo* or the mutation is recessive. With the exception of the monogenic forms of familial PD, which are frequently early-onset [12,13], familial and sporadic PDs are clinically indistinguishable [14,15]. Then, how do we go about modeling sporadic PD? Are the models for familial and sporadic PDs fundamentally different or based on similar strategies? To what extent do familial PD models provide relevant knowledge for sporadic cases and vice versa? This review aims to address these questions by introducing the literature, in which sporadic PD models were established or used for mechanistic or descriptive studies in *Drosophila*.

## 2. How to Model a Disease That Occurs Sporadically?

Sporadic PD, also referred to as idiopathic PD, is by definition a PD case whose cause is unknown. As such, it is intrinsically difficult to generate models through an approach that targets the “causal” genes or agents. Nevertheless, the recent progress in identifying candidate genetic and environmental risk factors for PD has made it possible to engineer animal models that carry genetic variations thought to increase the risk of PD, in the same way as most genetic models of familial PD were established. The analogous approach is the exposure to chemical agents known to be associated with an increased risk of PD. In addition, there may be a third category of sporadic PD models, which are developed by serendipity, such as the discovery of a mutant animal that exhibits a phenotypic similarity to PD. Furthermore, as sporadic PD is likely to be multifactorial and combinations among these strategies may result in a valid model.

On evaluating the value of animal models, it is worthwhile to perform a mental exercise imagining two extreme theoretical scenarios:

(A) A genetically engineered animal model perfectly recapitulates a known risk genetic variant for PD, but shows no characteristics similar to those of PD.
(B) A mutant animal was discovered to exhibit phenotypes similar to the cardinal characteristics of PD, but the mutant allele/gene is not known to be associated with PD risk.

Which model can be considered a model for sporadic PD?

In theory, an ideal model should possess similarities with the human disease in the (1) genetic basis; (2) pathological responses; (3) phenotypic endpoints; (4) underlying mechanism(s); and (5) response to known drugs with clinical efficacy. It should also be (6) predictive of human response to drugs [16]. However, there is a consensus in many disease research fields that no single animal model is expected to possess all these features. The two previously described scenarios exemplify this limitation. The lack of phenotypic similarity in (A) and known genetic association with PD in (B) may be viewed as weaknesses; however, given the heterogeneity and the multifactorial nature of sporadic PD, one can interpret the lack of phenotype in (A) as a missing interacting genetic or environmental factors for developing PD and speculate the existence of rare genetic variants similar to those in (B) in the human population. Thus, the apparent weakness can provide new opportunities for discovery that lead to a better understanding of the pathophysiology and underlying mechanisms of the disease. Therefore, I take a liberal position and propose to consider the animal models that possess any of the criteria (1) to (6) legitimate. On the basis of this premise, the current review recognizes animal models that possess one of the following features as sporadic PD models:

- Based on chemical or genetic agents known to be associated with PD risk
- Show functional impairments or loss of DA neurons, age-dependently
- Exhibit behavioral abnormalities similar to the movement disabilities in PD
- Responsive to symptomatic anti-PD medications, such as l-dopa
- Show Lewy body pathology

These models may be roughly classified into three categories by the methodology: (1) chemically induced models; (2) targeted genetic models; and (3) serendipitous genetic models. Approaches that combine multiple methods within and among these categories to create more realistic models are also emerging with success.

## 3. Why Use *Drosophila* for Parkinson’s Disease Research?

Various animal and cell culture models of PD have been created and have substantially contributed to understanding the disease. Both in vivo and in vitro models are broadly categorized into toxin-induced or genetic models. As previously discussed, although none of the existing models fully recapitulates all pathological features of PD, each model provides opportunities to investigate certain aspects of the disease. For example, cellular models are convenient to investigate protein aggregates associated with neuronal death and test compounds for therapeutic applications. However, pathogenic processes that involve interactions among different cell types and tissues can only be studied in animal models [17]. Primates and rodents are widely used to create in vivo mammalian models, which provide excellent tools to elucidate the biochemical and neuronal mechanisms of key pathological features of PD, such as protein aggregation, DA neuron demise, and behavioral abnormality. Recent progress in mouse genetic tools that enable cell type-specific and temporal control of gene expression has further advanced our understanding of the molecular genetic basis of PD pathogenesis. However, although theoretically possible, it is practically difficult to apply in vivo mammalian models for genetic and drug screens because of the cost, ethical concerns, and the animal’s lifespan, which is generally longer than 2 years [18,19].

*Drosophila melanogaster* is a model organism that provides numerous advantages for biomedical research. The fly genome is smaller with substantially less gene redundancy than that of mammals, while it contains homologs of approximately 70% of human disease-related genes [20]. Advanced genetic tools enable mutagenesis, silencing, and the overexpression of the gene-of-interest in a cell-type specific and temporally controlled manner [21]. Thus, research in flies has proven potential to discover conserved genetic pathways relevant for human disease [22]. Flies are amenable for large-scale genetic and chemical screening, thus providing opportunities for not only understanding the genetic and molecular basis of diseases but also for drug discovery [23]. Moreover, the relatively short life cycle and lifespan of flies (approximately 10 days and 2 to 3 months, respectively) accelerate the study of age-related disorders, including PD.

**Figure 1:**
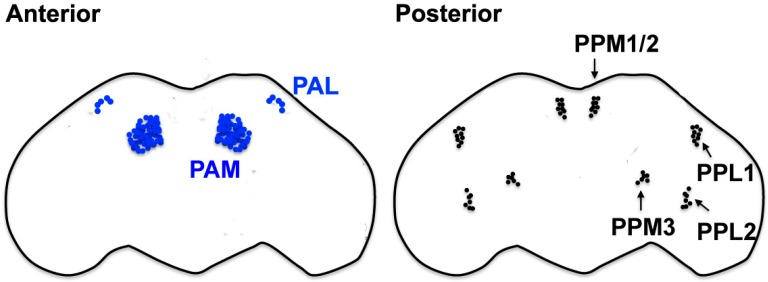
The dopaminergic (DA) neurons in the adult fly brain. Schematics of the seven major clusters of DA neurons located in the anterior and posterior brain [24,25,36]. The protocerebral anterior lateral (PAL) and protocerebral anterior medial (PAM) clusters reside in the anterior brain. Major posterior clusters include the protocerebral posterior medial 1, 2, and 3 (PPM1, PPM2, and PPM3, respectively) and the protocerebral posterior lateral clusters 1 and 2 (PPL1 and PPL2, respectively).

The fly’s nervous system is simpler but shares many features in structure, organization, and function with mammalian counterparts. Flies exhibit various DA-dependent behaviors controlled by different subclasses of DA neurons (Figure 1), whose individual roles and connectivity are unfolding [24,25]. The startle-induced climbing assay and its variations (such as startle-induced negative geotaxis; SING) are the most commonly used methods to quantify a fly’s locomotor behavior in comparing the organismal phenotypes of flies and human movement disorders [26,27,28,29]. These assays are distinct from recording the natural tendency to climb, referred to as negative geotaxis; however, they monitor the rate of the climbing response of flies when knocked down to the bottom of a cylinder. The climbing response is normally rapid, and a failure to recover from the “shock” of being knocked-down suggests defects in the functioning of the nervous system in controlling initiation. Noteworthy, recent studies have demonstrated that at least a subset of DA neurons in the protocerebral anterior medial (PAM) cluster has important roles in the climbing response, similar to DA neurons in the mammalian SNpc, which makes flies an ever-relevant model system for the investigation of PD [26,30,31].

The nonmotor symptoms of PD include olfactory dysfunction, mood disorders, cognitive impairments, sleep disorders, autonomic dysfunction, pain, and fatigue. These symptoms are common in early PD and are thought to be present prior to the onset of the motor symptoms [1]. Fly PD models have also been effectively used to investigate these non-motor symptoms, including sleep and circadian dysfunctions [32,33], visual deficits [34], and learning and memory abnormalities [33].

Despite these advantages, there are several limitations of *Drosophila* as a model organism to investigate PD. Flies are not the best organism for biochemical studies that require a large amount of homogeneous tissue. Flies do not have an adaptive immune system, which has important implications in the pathophysiology of PD [35]. Another notable limitation is the absence of a fly homolog of *SNCA*, the gene that encodes α-syn. Consequently, Lewy body pathology is not endogenously observed in flies. However, as subsequently discussed, the formation of Lewy body-like inclusions may be induced by the ectopic expression of human α-syn in flies [28], which indicates that the cellular machineries necessary for Lewy body formation and the pathophysiological impact of inclusions may be investigated in flies. In summary, *Drosophila* provides opportunities to rapidly discover the molecular-genetic mechanisms of PD and test therapeutics. Following up the discoveries made in flies with complementary studies using other animal and cellular models would be a promising path toward a comprehensive understanding of the disease and the development of new intervention strategies.

## 4. Chemically Induced Models of Sporadic PD

### 4.1. The Environmental Toxicology of PD

Epidemiological studies have suggested that several environmental factors, including pesticide exposure, rural living, well-water drinking, and agricultural occupation, increase the risk of PD [37]. Exposure to pesticides is the most consistently identified environmental risk factor for PD. A meta-analysis study reported a 62% increase in PD risk in a comparison of ever versus never pesticide exposure [38]. The pesticide exposure dose-dependently correlates with the risk of PD, which partly explains the increased risk in agricultural workers [39,40,41,42]. Although some inconsistency and heterogeneity among association studies have been reported [43,44], the idea to translate chronic pesticide use into animal models comes naturally. Indeed, the advent of the classic strategy to model sporadic PD using compounds found in pesticides dates before the expansion of the genetic models of PD.

Pesticides include insecticides, herbicides, and fungicides and encompass over 900 compounds that belong to many different chemical classes [45]. It is therefore tremendously difficult to determine which compounds pesticide users were exposed to. Consequently, only a small handful of chemicals have been used to develop PD models in animals. Rotenone and paraquat are among the most widely used.

Rotenone is a naturally occurring chemical found in the roots of tropical plants, commonly used as an insecticide. Being plant-derived and organic, it was originally thought to be harmless to humans when properly used. However, epidemiological studies have shown the increased risk for PD in rotenone users [46], and its use has been prohibited or restricted in many countries. It is a lipophilic compound that can freely cross the blood-brain barrier and cell membrane. The toxicity of rotenone is largely attributed to its effect on the inhibition of mitochondrial complex I activity [47], although other mechanisms, including microtubule depolymerization, may contribute to its toxicity [48].

Although not a common environmental toxin, MPTP (1-methyl-4-phenyl-1,2,3,6-tetrahydropyridin) was the first chemical that demonstrated the role of neurotoxins in the etiology of “Parkinsonism” (a set of symptoms typically associated with PD). MPTP is a byproduct of the synthesis of a meperidine analog MPPP (1-methyl-4-phenyl-4-propionoxypiperidine), a synthetic opioid drug and was found to be the causative agent of symptoms similar to advanced PD in intravenous drug users [49]. MPTP crosses the blood-brain barrier and is converted to the toxic 1-methyl-4-phenylpyridinium ion (MPP^+^) by monoamine oxidase B (MAO-B) in glia. Due to its high affinity to the dopamine transporter (DAT), MPP^+^ is selectively taken up by DA neurons. Thus, MPTP exposure causes rapid, nonprogressive DA neuron loss and associated motor disabilities in humans, as well as in various animal models [50].

Clinically, MPTP exposure is one of the exclusion criteria for PD because of the known consequence of this drug’s action, which is acute and nonprogressive Parkinsonism, unlike in chronic PD [1]. However, the discovery of MPTP boosted a search for its analogs, including paraquat, one of the most widely used herbicides. Paraquat is structurally similar to MPP^+^, can cross the blood-brain barrier (BBB) and is likely to be taken up to DA neurons as a form of paraquat^+^ [51]. It is an oxidative stressor that, by acting as a redox cycler, produces superoxide radicals and other reactive oxygen species (ROS). Paraquat also increases superoxide radical production in mitochondria [52,53]. Several case-controlled epidemiological studies have shown an increased risk of PD with paraquat exposure [46,54].

### 4.2. The Use of Rotenone and Paraquat to Model Sporadic PD and Its Variations

Chronic rotenone exposure is one of the first toxin-based fly models of sporadic PD. Flies fed with a sublethal dose of rotenone (<750 μM) for 7 days show impaired locomotor ability in startle-induced climbing assays and neuronal loss selectively in several subclasses of DA neurons. The administration of Levodopa (l-dopa), the most effective symptomatic medication for PD, partially rescues rotenone-induced climbing defects, but not a neuronal loss. l-dopa can cross the BBB, be taken up by DA neurons and converted to DA by dopa decarboxylase. These results indicate that chronic rotenone exposure induces the selective loss of DA neurons, which leads to reduced DA signaling and locomotor deficits and thus recapitulates the key characteristics of PD [27]. Using a slightly modified protocol, another group also showed that a 10-day rotenone (250 μM) exposure induces the loss of DA neurons in the PPL1 and PAL clusters, which progresses at least up to 6 weeks after the treatment [55].

Similar to rotenone, paraquat has been frequently used in different model animals to test its causal relationship with the pathological characteristics of PD. Several studies in flies have consistently demonstrated that chronic paraquat exposure causes a DA neuron loss or functional impairments and locomotor deficits within hours to days, accompanied by a high lethality. Feeding flies with 20 mM paraquat induces locomotor defects within 12 h and a neuronal loss selectively in several classes of DA neurons as early as 6 h after the treatment. Despite these rapid and drastic effects, it does not induce obvious brain-wide tissue damage, judging from the overall morphology of the brain and cholinergic neurons [56]. Similarly, DA neuron loss in the PPL1 cluster was observed 24 h after the exposure to 10 mM paraquat [55]. It was also shown that 20 mM paraquat ingestion induces pathological morphological changes within 24 h in several subclasses (PPL2, PPL2, PPM1/2, PPM3, PAL, and PP2) of DA neurons, such as the shrinkage of cell bodies, which is indicative of functional impairments [57,58].

These chemically induced models have successfully shown the relevance of environmental agents, in particular, the pesticides rotenone and paraquat, in DA neuron demise and motor disabilities, but not without criticism. In general, toxins fail to recapitulate the progressive nature of neurodegeneration in PD, although rotenone-induced progressive DA neuron loss is reported [55]. An inevitable drawback is lethal (they are pesticides, after all); depending on the concentration and application route, rotenone and paraquat kill flies within hours to days. The dose of rotenone or paraquat that is effective to induce DA neuron loss falls within the range where the median survival time is 7 days or less during chronic exposure [55]. Furthermore, there are several discrepancies among the results of different studies, which may originate from the subtle differences in the toxin administration protocols in the laboratory environment. The absence of DA neuron loss after the chronic exposure of 500 μM rotenone or 100 μM paraquat for 7 to 10 days has been reported [59]. A study found a morphological sign of declined viability in DA neurons in flies exposed to 500 μM rotenone; however, there were no aberrant changes in DA neurons in flies treated with 20 mM paraquat for 72 h [60].

Nevertheless, these apparent shortcomings can be considered evidence that supports the multifactorial origin of sporadic PD. The inconsistency in the results is indicative of the necessity of synergism with other chemicals or genetic variants for DA neuron demise and locomotor deficits. It is extremely difficult to measure the dose of environmental toxin exposure in humans; however, it is unlikely to be comparable to the lethal or sublethal dose in the toxin ingestion experiments in flies. Thus, more realistic models of sporadic PD may be created by combining multiple chemicals of noneffective drug doses or exposing single or multiple chemicals to the flies of different genetic backgrounds. The synergism of chemical agents and genetic factors can be tested on known candidate genetic variants or by screening for genetic modifiers that enhance or suppress the effect of the chemicals.

In line with this view, numerous studies have examined the synergism of multiple compounds or compounds and genetic factors on PD risk. Maneb (manganese ethylenebisdithiocarbamate) and ziram (zinc dimethylbisdithiocarbamate) are commonly used fungicides that belong to the class of dithiocarbamates. Epidemiological studies that evaluated the effect of ambient exposure to pesticides found an increased risk of PD associated with maneb or ziram alone and a greater risk with the combined use of paraquat, maneb and ziram [61,62]. In flies, chronic exposure to 4 mM paraquat, 500 μM maneb, or 1 mM ziram (the dose that does not decrease survival) does not individually impair DA neuron viability or climbing ability. In contrast, the combined exposure of paraquat and maneb, but not paraquat and ziram, leads to DA neuron loss in the PPL1 cluster at 6 weeks [55,63].

The approach to combine toxin exposure and genetic manipulations in flies is increasingly common and has uncovered important molecular insights into the gene-environmental interactions that increase or decrease PD risk. For example, heat-shock protein-70 (Hsp-70) and a mutation in the *methuselah* gene have been shown to ameliorate paraquat-induced PD-like phenotypes in flies [58,64]. One study showed that RNAi-mediated knockdown of the gene that encodes tyrosine hydroxylase (TH), the rate-limiting enzyme for DA synthesis, in several subclasses of DA neurons extends the lifespan and partially suppresses DA neuron loss in rotenone-treated flies (500 µM chronic exposure). These results suggest that DA production enhances the deleterious effects of rotenone on the viability of DA neurons and the lifespan [65]. Similarly, another study found that the overexpression of *Drosophila vesicular monoamine transporter* (*dVMAT*), which is required for packaging DA into synaptic vesicles, protects DA neurons from rotenone-induced loss, presumably by reducing the cytosolic DA pool [55]. These findings partly explain why DA neurons are selectively sensitive to rotenone and are concordant with the hypothesis that DA metabolism is a contributing factor in the selective vulnerability of SNpc DA neurons in PD [66].

In contrast, several studies indicate the opposite relationship of DA production and toxicity in paraquat models of PD; that is, an increase in DA production confers protection against paraquat-induced neurotoxicity. Loss-of-function mutations in the *Catecholamines-up* (*Catsup*) gene, which encodes a negative regulator of DA production, unexpectedly promote the survival and protect DA neurons from degeneration in paraquat-treated flies [56]. Because the overexpression of dVMAT, which leads to the reduction of the cytosolic DA pool, does not enhance or exacerbate paraquat-induced DA neuron loss [55], the increase in extracellular DA rather than cytosolic DA likely reduces paraquat-induced neurotoxicity. Parallel with these observations was the finding that overexpression of the TH gene in DA neurons or serotonergic neurons equally promotes resistance against lethality in paraquat-treated flies [57]. This study also investigated how the increase in extracellular DA counteracts the paraquat-induced neurotoxicity by correlating the expression of the D_1_-like dopamine receptor, DAMB, and toxicity. DA signaling via DAMB typically increases the cytosolic Ca^2+^ levels, which, in excess, enhance the oxidative stress and excitotoxicity. However, they found that TH overexpression causes a compensatory downregulation of DAMB in postsynaptic glutamatergic neurons and thereby confers resistance against paraquat-induced neurotoxicity [57].

Thus, rotenone-induced neurotoxicity may be chiefly mediated by a cell-autonomous mechanism sensitive to the cytosolic DA levels. In contrast, the systemic neurotoxicity of paraquat, including the selective loss of DA neurons, is dependent on the extracellular DA and mediated by non-cell-autonomous mechanisms. The reason for this intriguing mechanistic difference is unknown; however, it may be partly attributable to the toxin’s biochemical mechanisms of action. Both rotenone and paraquat cause oxidative damage, but through distinct molecular events. The toxicity of rotenone is mainly caused by the inhibition of mitochondrial complex I [67], which results in a decline of energy production by the respiratory chain and increased ROS production [68]. In contrast, paraquat has little or no effect on mitochondrial complex I [69], and its toxicity is thought to be essentially due to its redox cycling. This leads to the production of ROS in the form of hydrogen peroxide (H_2_O_2_) and hydroxyl radicals (HO) and the oxidation of NADPH, the major source of reducing equivalents in the cell [70].

A recent study by Stephano et al. [71] showed that chronic exposure to 500 µM rotenone does not cause a significant DA neuron loss for at least up to 10 days, whereas the climbing ability declines from day 6 onwards. They took advantage of the absence of neuronal loss and isolated DA neurons after 3 days of rotenone treatment, the timepoint considered to be the presymptomatic phase, and analyzed their transcriptome. A differential gene expression analysis that compared the DA-neurons of rotenone-treated and control flies showed the gene expression signature, which indicated the activation of the MAPK/EGFR- and TGF-β signaling pathways and the inhibition of the Wnt signaling pathway in rotenone-treated flies, including a reduced expression of *armadillo*/*β-catenin*, a central component of Wnt signaling. Concordant with this finding, the overexpression of *armadillo* in DA neurons ameliorates rotenone-induced locomotor decline, which suggests a role for Wnt signaling in the pathogenesis of PD [71]. Further implications of this work include the potential use of transcriptomic datasets to identify prodromal disease biomarkers.

Mitochondrial uncoupling protein 2 (UCP2) belongs to a class of the anion transporter family present in the inner mitochondrial membrane. UCP2 uncouples the mitochondrial electrical transport chain from the phosphorylation of ADP to ATP synthesis by dissipating the proton gradient across the mitochondrial inner membrane. Through this process, UCP2 attenuates ROS production and protects cells from oxidative damage [72]. The overexpression of human UCP2 (hUCP2) in several subclasses of DA neurons ameliorates the locomotor decline and partially prevents DA neuron loss induced by chronic rotenone exposure [73], likely by counteracting the mitochondrial damage induced by rotenone [74]. Although whether endogenous *Drosophila* UCPs are involved in rotenone-induced toxicity remains unaddressed, these findings suggest UCP2 as a potential therapeutic target for PD.

In summary, *Drosophila* provides opportunities to delineate the toxin-specific mechanism of action on DA neurodegeneration and can provide new insights into understanding the PD of diverse etiological origins. Furthermore, the powerful genetic toolbox of *Drosophila* enables the identification of genetic modifiers of toxin-induced neurodegeneration with relative ease. It is anticipated that the investigation of the synergism between toxins and genetic variants will be increasingly popular to validate the role of common genetic variants and their underlying mechanisms in PD susceptibility. The toxin-genetic combined approach might even discover new genes, whose cryptic variations normally have little effect on human health, but increase PD risk in certain environmental conditions.

## 5. Genetic Models of Sporadic PD

The identification of several causative genes for rare monogenic forms of PD, such as *SNCA* (*α-synuclein*) [75], *Leucine-rich repeat kinase 2* (*LRRK2*) [76], *Parkin* [77], *PTEN-induced putative kinase 1* (*PINK1*) [78] and *DJ-1* [79], overturned the traditional view that PD is not hereditary and unequivocally demonstrated the major role of genetics in PD etiology [80]. Furthermore, the past decade has seen considerable progress in identifying genetic risk variants associated with PD through linkage analysis or genome-wide association studies (GWAS). Recent GWAS meta-analyses have identified 41 loci, encompassing a total of 72 candidate genes, associated with sporadic PD risk [81,82]. Over 70% of the PD risk loci have protein altering or *cis*-QTL variants, which suggests that changes in the function or expression levels of candidate genes are associated with the increased PD risk (Table 1). Importantly, in agreement with previous studies, these studies found polymorphic variants of *SNCA* and *LRRK2* in association with sporadic PD. These studies also confirmed *MAPT* and *GBA* as PD-susceptibility factors [83].

The discovery of genetic factors implicated in PD risk has motivated the generation of genetic models in both vertebrate and invertebrate animals [18,84]. These models have been instrumental in advancing our understanding of the pathophysiology and molecular mechanisms of PD. In flies, the most common approach to generate genetic models is to introduce causal or disease-associated genetic variations in their fly homologs or express human genes in wild-type or mutant forms. The latter is applicable only for dominant mutations. These methodologies are used to model both familial and sporadic PD. Moreover, the genetic models based on the familial PD-linked genes that are also implicated in sporadic PD (i.e., *SNCA* and *LRRK2*) are often not specifically designated as sporadic PD models. This is in line with the view that the boundaries between familial and sporadic PD are increasingly blurred. The following sections will first discuss the targeted genetic models based on familial PD-causal genes, whose common variants are associated with sporadic PD risk. Second, the models developed based on the genetic susceptibility factors associated with PD will be introduced. Finally, the genetic models of sporadic PD developed through serendipity will be discussed.

### 5.1. Familial PD-Linked Genes in Sporadic PD: SNCA and LRRK2

One of the most notable discoveries with regard to the genetics of sporadic PD is that some low-penetrance variants of the familial PD-linked genes, *SNCA* and *LRRK2*, are also present in sporadic cases [7,83,85]. *SNCA*, which encodes α-syn, is a causal gene of autosomal dominant familial PD. At least five pathogenic mutations (A30P, E46K, A53T, H50Q, and G51D), as well as gene duplications and triplications have been identified in approximately 1% of PD families [75,86,87,88,89,90]. *SNCA* dominant mutations are extremely rare; however, the discovery that α-syn is the major constituent of Lewy bodies [3], the pathological hallmark of most familial and sporadic PD, prompted the search for *SNCA* polymorphisms in sporadic cases. Duplications have been found in apparently sporadic cases [91,92], as well as in asymptomatic carriers [91,93], with an estimated penetrance of approximately 40% [91,94]. These findings suggest the presence of other genetic modifiers or environmental factors in the pathogenesis of PD due to SNCA duplication. An association of PD risk with polymorphisms in the promoter region and 3′ UTR of the SNCA gene is also reported [95,96,97]. The duplications and variants likely increase the expression levels of α-syn protein, thereby contributing to PD pathogenesis [98,99,100]. A recent work that evaluated the SNPs in the cis-regulatory elements of the *SNCA* gene on the gene expression levels and pathogenesis broadly confirms this conclusion [101].

α-Syn is abundantly expressed in the nervous system and enriched in the presynaptic terminals [102]. It is highly soluble and unfolded in aqueous solutions; however, it can self-assemble to form fibrils. Multiple lines of evidence suggest that the soluble prefibrillar form of α-syn oligomers, rather than larger aggregates, are the toxic species mediating the cellular dysfunction and degeneration [103]. α-Syn toxicity is rooted in its propensity for oligomerization, and disease-causing mutants are more prone to oligomerization than wild-type α-Syn [103]. Recent studies have also shown that α-syn oligomers can be released from neurons and spread in a prion-like manner by seeding with the soluble α-syn of the recipient cells [104,105,106,107].

Although *Drosophila* does not have *SNCA* homologs, because genetic and molecular evidence indicates that toxic gain-of-function mechanisms underlie α-syn-linked PD, the transgenic overexpression of human α-syn in flies provides a useful tool to investigate the pathogenesis of α-syn-linked PD. The overexpression of the human wild-type or mutant form of α-syn was the first PD model ever created in flies [28]. Similar strategies have been used in numerous studies to date and have shown that the overexpression of wild-type or mutant human α-syn impairs the function or survival of DA neurons and causes PD-like locomotor deficits [28,30,108,109,110,111], although some discrepancies exist regarding its effect on DA neuron loss [60,112,113].

Mutations in *LRRK2* are the most frequent cause of PD, found in approximately 10% of autosomal dominant familial PD and 4% of sporadic cases worldwide [114]. The most common mutation, G2019S, is found in 4% of familial and 1% of apparently sporadic cases. The estimated penetrance of the G2019S mutation ranges from approximately 30 to 70%, depending on the age and ethnic background [115]. The G2019S mutation is particularly frequent in sporadic PD patients among Ashkenazi Jews (13%) [116] and North African Arabs (41%) [117]. No disease case caused by *LRRK2* deletion or multiplication has been reported, and the disease phenotypes of homozygous *LRRK2* mutant carriers are similar to those of heterozygous carriers [118]. Overall, these findings suggest that *LRRK2* G2019S manifests PD by gain-of-function mechanisms that involve interactions with other genetic or environmental factors.

*LRRK2* encodes a large 2527 amino-acid protein that contains multiple conserved domains, including a GTPase domain, a kinase domain, Armadillo, an Ankyrin repeat, a leucine-rich repeat, MAPKKK (MAP kinase kinase kinase), and WD-40 domains [119]. The G2019S mutation resides in the MAPKKK domain, and the mutation increases the kinase activity by at least two- to three-fold in vitro [120,121]. *Drosophila LRRK* (*dLRRK*) is the unique homolog of human *LRRK2*. Consistent with the notion that LRRK2 gain-of-function mutations cause PD, *dLRRK* null mutant flies show little or no defects in the viability of DA neurons [122], although some controversy exists [123]. Numerous transgenic fly lines expressing the wild-type or mutant form of *dLRRK* or human *LRRK2* have been generated in an attempt to model dominantly inherited PD, with varying consequences in neuronal loss and locomotion. Pan-neuronal or DA neuron-targeted expression of human *LRRK2* G2019S mutants, the genetic model relevant for sporadic PD, causes the selective age-dependent loss of DA neurons, locomotor deficits, and premature mortality [124,125,126,127,128].

Given that there are many excellent reviews summarizing the findings on α-syn or LRRK2 models in flies (such as [129,130,131,132]), instead of listing findings from individual papers, this review elects to briefly discuss the overarching insights from a large body of work based on these models. One of the emerging notions is the diversity of the mechanisms underlying the DA neuron demise. To date, there is no single unified theory explaining the pathogenic mechanism of α-syn overexpression or *LRRK2* G2019S mutation; however, various intracellular machineries are implicated. Studies using *Drosophila* models have established a bewildering list of mechanisms involved in the α-syn pathogenicity. These mechanisms include alterations in the endoplasmic reticulum (ER)-Golgi trafficking and synaptic vesicle formation [133,134], glutathione abundance [111], glucose metabolism [135], actin cytoskeleton that leads to impaired mitochondrial dynamics [110], mitochondrial integrity and function [136], macroautophagy [137], and elevated oxidative stress levels [138,139,140].

An increasing number of potential LRRK2 phosphorylation targets involved in PD pathogenesis has been identified in the studies that used the fly model of *LRRK2*-linked PD, including the glycogen synthase kinase 3b (GSK 3b) [125], microtubule-binding protein 1B (MAP1B) homolog Futsch [141], endophilin A (EndoA) [142,143], eukaryotic initiation factor 4E-binding protein (4E-BP) [124], and ribosome protein s15 [144]. The phosphorylation of these targets by LRRK2 dysregulates cellular processes, such as dendritic and synaptic morphology, synaptic endocytosis, macroautophagy, and translational machinery. In addition, genetic and functional interaction assays have indicated several factors involved in the neurotoxicity of LRRK2, such as vacuolar protein sorting 35 (VPS35) [145,146], RAB7L1 [147], and ArfGAP1 [148,149], which are involved in vesicular trafficking, retromer, the lysosomal pathway, and miRNA-mediated translational inhibition.

The discovery of these numerous targets and pathways leads us to the next important questions: how are the multiple pathways related? Do they additively or synergistically act to disrupt cellular homeostasis, thereby leading to the eventual cell death? One connecting point may be the mitochondrial dysfunction, which has been proposed as a central pathogenic mechanism of PD in an increasing number of studies [150]. A healthy mitochondrial network is maintained by multiple quality control mechanisms, including the fission-fusion dynamics and clearance of damaged mitochondria via the autophagy-lysosome pathway, termed mitophagy [151]. Studies in flies have demonstrated that regulators of mitophagy, including the *sterol regulatory element binding transcription factor 1* (*SREBF1*) gene known to be associated with the risk of sporadic PD, modulate the DA neuron toxicity caused by the mutations in familial PD-linked genes, *PINK1*, *parkin*, and *LRRK2*. These studies contribute evidence to the notion that sporadic and monogenic familial PD have common pathological mechanisms that converge on the abnormal mitochondrial homeostasis and mitophagy dysfunction [152,153,154].

Given the clinical, geographic, and ethnic heterogeneity of PD patients, it is also possible that different pathogenic mechanisms are triggered depending on the genetic background, environmental conditions, or in a state-dependent manner. To date, there is no clear view as to why the same mutation (e.g., *LRRK2* G2019S) is causal to both familial and sporadic PD. Understanding whether and how different pathogenic mechanisms interact will help comprehend the mechanistic differences between familial and sporadic PD, for which *Drosophila* models can provide useful tools.

Both α-syn and *LRRK2* G2019S models are based on overexpression via the use of binary systems, such as GAL4/UAS and LexA/LexAop [21]. Although the results obtained by the misexpression of foreign genes must be interpreted with a grain of salt, carefully controlled misexpression experiments can be useful to analyze the cell-type specificity of toxicity and neuroanatomical correlates with behavioral phenotypes, as well as to distinguish cell-autonomous from noncell-autonomous consequences of overexpressed genes. A prime example is the use of α-syn overexpression to map the neuronal subtypes relevant for climbing defects, which identified a subgroup of DA neurons within the PAM cluster, thus suggesting functional parallels between this group of neurons with DA neurons in the SNpc [30]. Another example is the overexpression of *LRRK2* G2019S in DA neurons, which leads to noncell-autonomous degeneration in the visual system and the loss of visual function [155]. With the use of advanced genetic tools that enable gene expression in a spatially and temporarily tunable fashion, it is likely that the misexpression strategy will provide further insights into the mechanisms that underlie PD associated with gain-of-function mutations.

### 5.2. Models Based on Known Genetic Risk Factors: GBA, MAPT, and GAK

#### 5.2.1. *GBA*

Homozygous or compound heterozygous mutations in the *GBA* gene that encodes β-glucocerebrosidase (GCase) cause Gaucher disease. Gaucher disease is a rare lipid storage disorder, and these patients are known to have an increased risk for PD. Moreover, single heterozygous mutations are associated with an increased risk of PD [7,81,83]. Approximately 300 mutations in *GBA* have been found to cause Gaucher diseases. Including the two common mutations associated with PD, N370S, and L444P, *GBA* mutations are considered to be loss-of-function mutations that lead to a deficiency in GCase. GCase is a lysosomal enzyme synthesized in the ER that undergoes N-linked glycosylation and folding and subsequently traffics to lysosomes through the Golgi. *GBA* mutations result in the accumulation of the lipid substrates of GCase [156]. Furthermore, the accumulation of misfolded mutant GCase causes ER stress and triggers the unfolded protein response (UPR). The resulting accumulation of misfolded protein, including that of α-syn, is thought to increase the risk of PD [157,158].

*Drosophila* has two homologs of the *GBA* gene, *dGBA1a*, and *dGBA1b*, both of which have approximately 50% similarity to GCase. Given that disease-causing GBA variants are loss-of-function alleles, several *dGBA1a/b* gene knockout or knockdown models that inactivate either or both of the *dGBA1* genes have been generated [159,160,161,162]. In addition, the finding that many mutations, including PD-associated N370S and L444P, are likely to be dominant-negative has led to the creation of transgenic flies that express wild-type or mutant human *GBA* [158,163,164]. The phenotypes of these loss-of-function and misexpression models are roughly in agreement in that they show a reduced lifespan, locomotor impairments, protein aggregation, and neurodegeneration. The *dGBA1a* gene expression is restricted in the midgut, whereas *dGBA1b* is ubiquitously expressed [165]. This finding explains the observation that *dGBA1a* deletion is not detrimental, whereas the *dGBA1b* loss-of-function leads to the deleterious phenotypes [159,166].

In contrast, the results are less consistent with regard to DA neuron loss in the *dGBA1* models. Some studies have indicated the loss of adult DA neurons [161,163,164], whereas other studies did not show DA neuron loss, although brain vacuolization was observed in *dGBA1* loss-of-function models [159]. One study reported that in flies expressing α-syn, the *dGBA1b* mutation increases the misfolding of α-syn and mildly enhances α-syn-dependent DA neuron loss; however, other phenotypes of the *dGBA1b* mutation are unaffected by the presence of α-syn. Therefore, DA neuron degeneration is primarily the consequence of α-syn overexpression, and α-Syn and *dGBA1* do not synergistically impair neuronal integrity [159], although a recent study contradicts this conclusion [167]. The mechanism regarding how *dGBA1* deficiency promotes abnormal protein aggregation is also under debate. One study proposed that *dGBA1b* deficiency causes autophagy defects that lead to the accumulation of protein aggregates [166], whereas another report found no evidence of autophagy defects, but an increase in extracellular vesicles, which is thought to spread the protein aggregates in the *dGBA1b* null mutants [168].

Although these differences warrant further studies to clarify the contribution of *dGBA1* in DA neuron degeneration and its underlying mechanisms, fly models have been effectively used to demonstrate that small molecule pharmacological chaperones that cross the BBB, ambroxol, and isofagomine, suppress GCase misfolding, reduce ER stress, and ameliorate the *GBA* mutant phenotype in vivo. These results provide supporting evidence that ER stress is a pathogenic process in *GBA* mutants and suggest a therapeutic potential of small molecule chaperons in GBA-associated PD [161,163]. Collectively, the *dGBA1* mutation and *hGBA* misexpression models provide useful tools to better understand the molecular mechanisms that underlie the pathogenic process caused by *GBA* mutations and develop disease-modifying therapeutics.

#### 5.2.2. *MAPT*

The *MAPT* gene encodes the microtubule-associated protein tau essential for tubulin polymerization and microtubule stability [169]. Accumulation of the misfolded tau protein is a pathological characteristic of a spectrum of neurological disorders referred to as tauopathies, including Alzheimer’s disease, progressive supranuclear palsy, corticobasal degeneration, and frontotemporal dementia. Although the majority of tauopathies occur sporadically, more than 50 pathogenic *MAPT* mutations were identified in approximately 150 families with tauopathies [170]. Tauopathies associated with *MAPT* mutations include familial and sporadic frontotemporal dementia (FTD) [171,172] (also referred to as “frontotemporal lobar degeneration with *MAPT* mutation (FTLD-tau)”) [173]. Intriguingly, Parkinsonism is a common comorbidity in patients of familial and sporadic tauopathies, and more than 50% of patients of Alzheimer’s disease manifest Lewy body pathology [174]. Furthermore, numerous studies, including GWAS meta-analyses, have confirmed the association of the *MAPT* locus and an increased PD risk in Caucasian populations [81,175]. These findings support the emerging view that tauopathies and synucleinopathies (associated with α-syn inclusions), the two disease classes characterized by the accumulation of pathological protein aggregates, belong to a continuum of disorders with significantly overlapping clinical features and pathogenic mechanisms [176].

The *MAPT* locus lies within a genomic region that has two conserved haplotypes, H1 and H2, defined by numerous single nucleotide polymorphisms [177]. H1 and H2 haplotypes cover the entire *MAPT* gene, differ in orientation and do not recombine [178] as they originate from a 900 kb inversion [179]. The H1 haplotype of the *MAPT* gene is associated with a risk of PD [180,181], as well as in sporadic tauopathies [182]. It has been shown that the H1 haplotype has a greater expression of the tau protein than H2 [183,184], and the H2 haplotype has a protective effect in neurodegeneration [185].

*Drosophila tau* (*dtau*) is a unique homolog of the *MAPT* gene. Given the central role of tau in tauopathies, it is not surprising that much of the related work on the fly is based on the overexpression of *Drosophila* or the human tau protein in wild-type or mutant forms. These models have been effectively used to investigate the toxicity of tau inclusions and their underlying mechanisms at the cellular and organismal levels (reviewed in References [186,187]). Deletion of the *tau* gene is not detrimental by itself and does not increase the toxicity of human amyloid-β on the fly’s lifespan [188], although *tau* deletion is reported to decrease the axonal microtubule number and density in larval and adult nervous systems [189]. One study demonstrated that, by ectopically expressing human tau and α-syn in the retina, DA neurons, and larval neuromuscular junction, tau overexpression enhances α-syn-mediated DA neuron loss. Conversely, α-syn overexpression enhances tau-induced motor dysfunction, microtubule disorganization, and axonal transport defects, among other neuronal dysfunctions [190]. These results are in agreement with the existence of the interaction between tauopathy and synucleinopathy and provide insights into the mechanism that underlies the role of tau in PD pathogenesis. Because the H1 haplotype is very polymorphic and includes subhaplotypes, defining specific genetic variants that associate with the increased risk of PD remains a major challenge [191,192]. Thus, fly models based on the polymorphisms in the *MAPT* gene associated with PD risk, rather than overexpression, are yet to be generated.

#### 5.2.3. *GAK*

A chromosomal locus that contains the *GAK* gene has been repeatedly found in GWAS [175,193]. Like many other genetic risk factors identified in GWAS, the association of *GAK* with PD risk may depend on the ethnic background and is replicated mainly in Caucasian populations [81,194]. *GAK* encodes cyclin-G-associated kinase, the association partner of cyclin-G and CDK5 and is involved in diverse cellular mechanisms, including clathrin-mediated endocytosis and mitotic progression [195]. The PD-associated *GAK* gene polymorphism reduces the GAK protein levels and correlates with the increase in α-syn expression [196]. A recent work demonstrated that RNAi-mediated silencing of *auxilin* (*aux*), the fly homolog of *GAK*, in DA neurons leads to age-dependent locomotor impairments, a reduced lifespan and progressive neuronal loss in several subclasses of DA neurons. Moreover, *aux* RNAi accelerates the onset and progression of DA neuron loss caused by α-syn overexpression [197]. These results support that *GAK* is a PD risk factor and *aux* gene silencing in flies provides a valid model to investigate the function of *GAK* in PD pathogenesis, including the potential synergism with α-syn toxicity.

### 5.3. Serendipitous Fly Models of Sporadic PD: Fer2 and Dfoxo Mutants

To date, only a few fly models that provide tools to investigate sporadic PD have been generated by serendipity. This may seem like an unusual path to model a human disease, compared to the targeted approach of using knowledge regarding the genetic and chemical factors relevant to the disease. However, historically, many animal models have been developed by serendipity, typically through the careful phenotypic analysis of mutant animals carrying spontaneous mutations or induced by forward genetics, and have contributed to the study of diseases [198]. This approach has the potential to fill the knowledge gap in the genetic factors involved in the disease pathogenesis. Using a statistical model termed genome-wide complex trait analysis (GCTA), which quantifies the “missing heritability” in PD, it was estimated that genetic factors could explain up to 27% of PD cases. In contrast, only 3–5% of cases have been attributed to the single nucleotide polymorphisms (SNPs) identified in GWAS [199]. This finding suggests that many more genetic variants that influence PD risk and could represent novel therapeutic targets may have yet to be discovered. The bottom-up approach transitioning from mutant phenotypic characterization to gene discovery has the potential to open a new avenue in PD research.

The bHLH-transcription factor *Fer2* (p48-related 2) in *Drosophila* shares a high homology with the mammalian transcription factors *p48/ptf1a* and *Ferd3l* (*Nato3*) within the bHLH domain. Both *p48/ptf1a* and *Ferd3l* are involved in the control of neuronal development [200,201,202]. In the fly brain, *Fer2* is expressed in a limited number of neurons, including a subgroup of circadian pacemaker neurons [203,204,205] and two subclasses of DA neurons, PAM and PAL [26,206]. Intriguingly, hypomorphic mutants of *Fer2* exhibit PD-like phenotypes typical for fly models of PD. These phenotypes include locomotor deficits that can be improved by l-dopa administration, a reduced lifespan and a progressive DA neuron loss in the PAM cluster. Because fewer numbers of PAM neurons are born in both strong and milder hypomorphic mutants of *Fer2*, the role of *Fer2* in PAM neuron development is indisputable. However, the knockdown of *Fer2* restricted in adulthood demonstrated that *Fer2* plays an essential role in the survival of adult DA neurons independent of its role in the development. Furthermore, it was also shown that oxidative stress elicited by the oxidants H_2_O_2_ or paraquat induces DA neuron loss specifically in PAM neurons in *Fer2* mutant flies. At the cellular level, the *Fer2* mutation causes abnormalities in the mitochondrial morphology and autophagosome formation, the well-known pathogenic molecular events implicated in PD, which suggests that *Fer2* controls the expression of genes involved in multiple mechanisms that are normally protective of DA neurons [26,207]. Collectively, these results suggest that *Fer2* mutant flies are useful for investigating the mechanisms of DA neuron demise, particularly the gene-environmental interaction with the presence of oxidative stress, thereby providing an opportunity to investigate pathogenic processes in PD.

The forkhead box family of transcription factors (FOXOs) are regulators of diverse biological pathways, including the stress response, metabolism and cellular homeostasis and are implicated in numerous diseases [208]. *Drosophila* has a unique homolog of FOXO, encoded by the *dfoxo* gene. Although no direct association of polymorphisms in FOXO factors with PD risk has been identified in the human population, changes in the gene expression levels in PD patients [209] and studies in rodents and flies suggest the role of FOXO factors in the survival of DA neurons in aged animals and PD models [210,211,212,213,214,215,216]. In addition, it was recently found that *dfoxo* null mutants and *Fer2* mutants are phenotypically similar, showing the selective loss of PAM cluster DA neurons, locomotor deficits, and a reduced lifespan. Both *Fer2* and *dfoxo* mutants exhibit mitochondrial damage and an autophagy defect specifically in PAM neurons. A genetic interaction analysis showed that the loss of function of *Fer2* and *dfoxo* additively, but not synergistically, aggravates DA neuron loss. Consistent with the finding that mitochondria and autophagy are commonly affected in both mutants, the overexpression of one gene can rescue the DA neuron loss caused by the mutation of the other gene. Interestingly, however, in contrast to *Fer2* mutants, oxidative stress does not enhance the DA neuron loss in *dfoxo* mutants. These results suggest that the activities of *Fer2* and *dfoxo* are regulated by different cellular stressors; however, their downstream pathways converge onto the process that leads to mitochondrial biology and autophagy, thereby promoting the survival of DA neurons in the PAM cluster [207].

The results of *Fer2* and *dfoxo* mutants highlight the complex interaction between multiple genetic and environmental factors that affect DA neuron viability and its impact on the pathogenesis of PD. It will be of substantial interest to determine whether their homologs, downstream or interacting genes in humans have a causal role in PD pathogenesis.

## 6. Conclusions

An increasing body of work to model sporadic PD in flies has verified candidate environmental and genetic risk factors and provided insights into their roles in the pathogenesis of PD. The current list of genetic risk variants is by no means exhaustive to explain all sporadic PD cases, and as the expansion and technological improvement of GWAS continues, many more susceptibility loci are expected to be identified and prompt the generation of genetic animal models of sporadic PD in the coming years. Furthermore, more unbiased approaches going from phenotype to genotype, including forwarding genetic screens, remain valuable to identify novel genetic factors that modify the risk or severity of PD. Fly models continue to provide effective tools to better understand whether and how different molecular pathways involved in the degenerative process interplay, which is the knowledge necessary to develop an efficient and potentially personalized treatment strategy for this complex disorder.

## Figures and Tables

**Table 1:** The candidate genetic risk factors for Parkinson’s disease (PD) identified in a recent genome-wide association study (GWAS) meta-analysis. The table is adapted from [81]. Genes in bold font are also linked to the monogenic forms of familial PD.

Chromosome	Gene
1	*GBA*, *NUCKS1*, *SLC41A1*, *ITPKB*, *SIPA1L2*
2	*IL1R2*, *TMEM163*, *CCNT2*, *SCN3A*, *STK39*
3	*CHMP2B*, *MCCC1*, *SATB1*, *NCKIPSD*, *CCDC71*, *ALAS1*, *TLR9*, *DNAH1*, *BAP1*, *PHF7*, *NISCH*, *STAB1*, *ITIH3*, *ITIH4*
4	*TMEM175*, *DGKQ*, *FAM200B*, *CD38*, *FAM47E*, ***SNCA***, *ANK2*, *CAMK2D*
5	*ELOVL7*
6	*ZNF184*, *HLA−DRB6*, *HLA−DQA1*
7	*KLHL7*, *NUPL2*, *GPNMB*
8	*MICU3*, *CTSB*, *SORBS3*, *PDLIM2*, *C8orf58*, *BIN3*
9	*SH3GL2*
10	*FAM171A1*, *BAG3*
11	*DLG2*, *MIR4697*
12	***LRRK2***, *OGFOD2*
14	*GCH1*, *TMEM229B*, *GALC*
15	*VPS13C*
16	*ZNF646*, *KAT8*, *COQ7*, *TOX3*
17	*ARHGAP27*, *CRHR1*, *SPPL2C*, *MAPT, STH*, *KANSL1*, *ATP6V0A1*, *PSMC3IP*
18	*SYT4*
19	*LSM7*
20	*DDRGK1*

## Abbreviations

PD	Parkinson’s disease
DA	dopamine
SNpc	substantia nigra pars compacta
α-syn	α-synuclein
SING	startle-induced negative geotaxis
PAM	protocerebral anterior medial
MPTP	1-methyl-4-phenyl-1,2,3,6-tetrahydropyridin
MPPP	1-methyl-4-phenyl-4-propionoxypiperidine
MPP^+^	1-methyl-4-phenylpyridinium ion
MAO-B	monoamine oxidase B
DAT	dopamine transporter
BBB	blood brain barrier
ROS	reactive oxygen species
Maneb	manganese ethylenebisdithiocarbamate
ziram	zinc dimethylbisdithiocarbamate
Hsp-70	heat-shock protein-70
PAL	protocerebral anterior lateral
PPM	protocerebral posterior medial
PPL	protocerebral posterior lateral
dVMAT	*Drosophila* vesicular monoamine transporter
Catsup	Catecholamines-up
DAMB	D_1_-like dopamine receptor
TH	tyrosine hydroxylase
H_2_O_2_	hydrogen peroxide
HO	hydroxyl radicals
UCP2	uncoupling protein 2
LRRK2	leucine-rich repeat kinase 2
PINK1	PTEN-induced putative kinase 1
GWAS	genome-wide association study
MAPKKK	MAP kinase kinase kinase
GSK 3b	glycogen synthase kinase 3b
MAP1B	microtubule-binding protein 1B
EndoA	endophilin A
4E-BP	eukaryotic initiation factor 4E-binding protein
VPS35	vacuolar protein sorting 35
SREBF1	sterol regulatory element binding transcription factor 1
GCase	β-glucocerebrosidase
ER	endoplasmic reticulum
UPR	unfolded protein response
FTD	frontotemporal dementia
GCTA	genome-wide complex trait analysis
SNP	single nucleotide polymorphism
Fer2	p48-related 2
